# Addressing Label Sparsity With Class-Level Common Sense for Google Maps

**DOI:** 10.3389/frai.2022.830299

**Published:** 2022-03-16

**Authors:** Chris Welty, Lora Aroyo, Flip Korn, Sara M. McCarthy, Shubin Zhao

**Affiliations:** Google Research, New York, NY, United States

**Keywords:** map, knowledge graph, crowdsourcing, class-level attributes, common sense, knowledge acquisition

## Abstract

Successful knowledge graphs (KGs) solved the historical knowledge acquisition bottleneck by supplanting the previous expert focus with a simple, crowd-friendly one: KG nodes represent popular people, places, organizations, etc., and the graph arcs represent common sense relations like affiliations, locations, etc. Techniques for more general, categorical, KG curation do not seem to have made the same transition: the KG research community is still largely focused on logic-based methods that belie the common-sense characteristics of successful KGs. In this paper, we propose a simple yet novel three-tier crowd approach to acquiring *class-level attributes* that represent broad common sense associations between categories, and can be used with the classic knowledge-base default & override technique, to address the early *label sparsity problem* faced by machine learning systems for problems that lack data for training. We demonstrate the effectiveness of our acquisition and reasoning approach on a pair of very real industrial-scale problems: how to augment an existing KG of places and offerings (e.g. stores and products, restaurants and dishes) with associations between them indicating the availability of the offerings at those places. Label sparsity is a general problem, and not specific to these use cases, that prevents modern AI and machine learning techniques from applying to many applications for which labeled data is not readily available. As a result, the study of how to acquire the knowledge and data needed for AI to work is as much a problem today as it was in the 1970s and 80s during the advent of expert systems. Our approach was a critical part of enabling a worldwide *local search* capability on Google Maps, with which users can find products and dishes that are available in most places on earth.

## 1. Introduction

From the outset, knowledge graphs (KGs) have prominently used crowdsourcing for knowledge acquisition, both from the perspective of scaling out graph creation and long-term maintenance, solving the historical knowledge acquisition bottleneck by revisiting the expert systems assumption that knowledge should be acquired from experts. As a result, popular KGs like Freebase (Bollacker et al., 2008)—now Google's Knowledge Graph—and WikiData (Vrandečić and Krötzsch, 2014) are composed primarily of popular “common sense” entities and relations in the world that people are exposed to regularly and that can be acquired from and validated by the crowd.

Similarly, today Google Maps overlays data on maps about the different places or establishments (stores, restaurants, hospitals, etc.) worldwide, and crowdsourcing plays a central role in the acquisition and maintenance of this information, as discussed in Lagos et al. (2020). Users contribute opening hours, locations, reviews, etc., as well as categorical information about places such as whether it is a supermarket, department store, etc., which makes KGs a natural representation for this information.

Despite such heavy and widespread success of KGs for representing entities in the world and their properties, Taylor (2017) points out that there has not been much attention paid in the research community to *class-level attributes* in KGs: graph edges between nodes that represent categorical terms, what they might mean and how to acquire them. For the purposes of this paper we use the words *type, category, class* interchangeably, as well as *attribute, property, relation*. Practical and industrial KG edges remain almost exclusively at the instance level (e.g., McDonalds serves Big Mac), and a few KGs may encode class-level domain/range constraints (e.g., Restaurants serve Food), but no KG includes attributes of classes that represent our common-sense knowledge about them (e.g., Burger Joints serve burgers). There has certainly been a lot of research published in the sub-fields of Knowledge Representation on axiomatic knowledge acquisition, for example Ji et al. (2020), but these methods are not well-suited for crowdsourcing and have not made the transition to any industrial KG settings.

**Figure 1:**
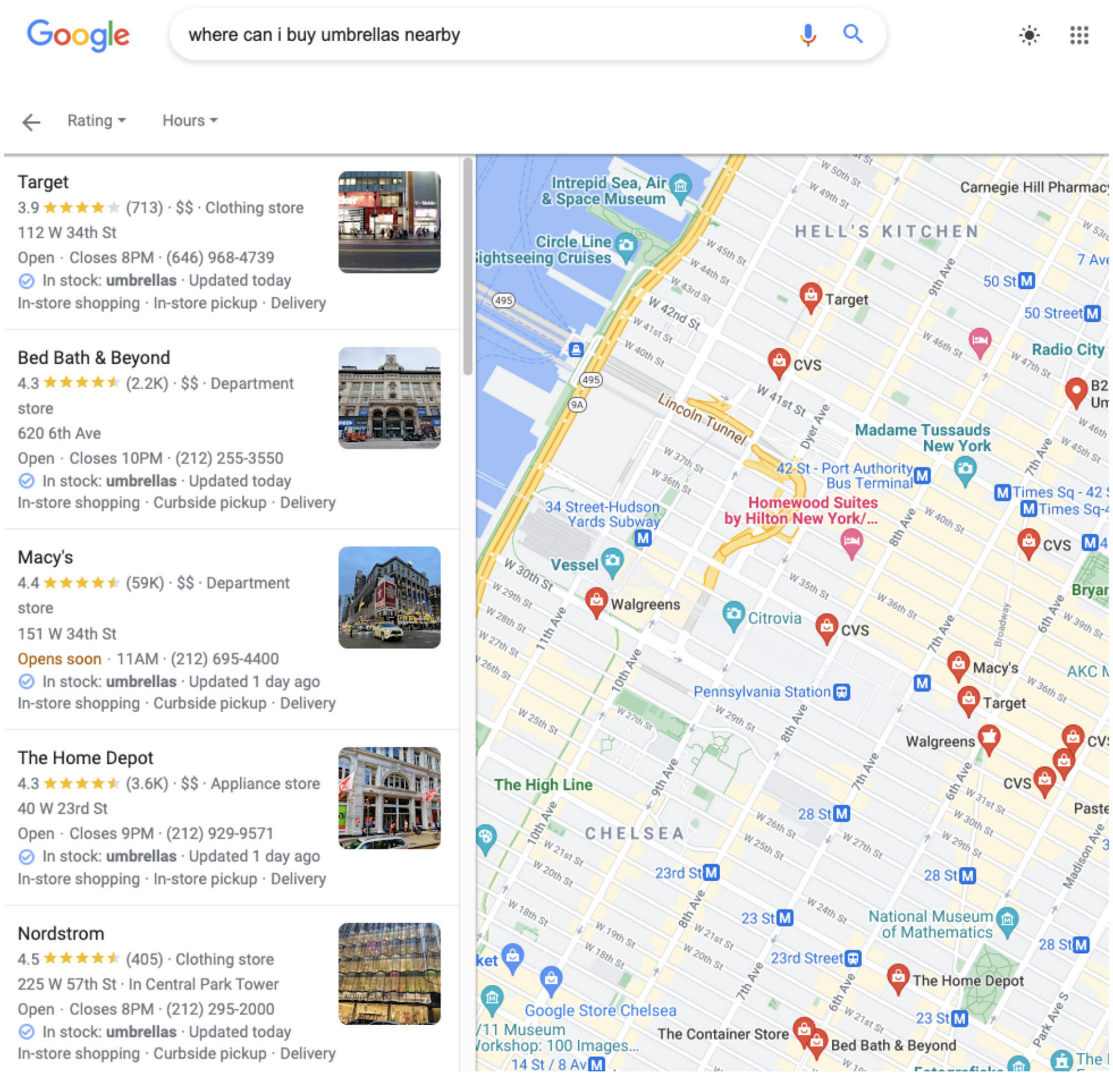
Google Maps local shopping search results for umbrellas in NYC shows stores that sell them.

In this paper we explore the question of acquiring common sense class-level attributes from the crowd and applying those attributes effectively with other sources of information to solve a knowledge-base completion (KBC) problem, as defined in Bordes et al. (2013), where success is measured by the precision and recall of graph edges. We take a particular problem, that of understanding what is *offered* at each *establishment* on earth. Such a KG could be used to answer questions like, “Where can I buy an umbrella nearby?” (see Figure 1), “Where can I eat lamyun?”, or “Where can I get a flu shot?”, etc. We call this problem *local offerings* and it is one that is of interest to search engines like Google.^1^

Local offerings, compared to on-line, poses a significant practical knowledge acquisition problem because real-world transactions do not occur on-line or the data is heavily siloed, and therefore data about what products are being sold at what stores, or what dishes are served at what restaurants, is not broadly available; it is a sort of “dark matter” of the web—we know it's there but can't directly observe it. Although it may seem familiar to us—e.g., brick and mortar shops that support on-line ordering and in-store pickup—such exceptions are actually quite rare, by the numbers. Less than 30% of stores worldwide having a website and even fewer that include a product catalog.^2^

Indeed, our data shows that web pages and merchant feeds account for less than 0.001% of the total matrix of products at stores. To address this shortage of web information, we harness the crowd in three tiers: *users* around the world who have visited stores and voluntarily provide instance-level product availability (e.g., Ajay Mittal Dairy sells Milk); a much smaller set of *paid raters* who curate class-level attributes connecting common sense store and product categories (e.g., Grocery Stores sell Milk); and a very small set of *paid operators* who call stores to confirm the instance-level associations as evaluation ground-truth labels. The intuition behind this combination is that a lot of the instance-level associations are obviously true or false at the categorical level, and that acquiring knowledge at that level can jump-start the instance-level acquisition and help it be more productive: don't waste a user's efforts answering about milk or asphalt at an individual grocery store when simple common sense tells us the answer. Due to the prominence of common sense curation in our approach, we call the project *CrowdSense* (CS).

To our knowledge, acquiring class-level attributes from the crowd in order to jump-start a KBC problem has not been attempted before, and there are very few examples of KBC problems at this scale (tens of millions of stores wordwide and more than 10k products). The project and approach led to a successful worldwide launch of local shopping results overlaid on Google Maps, and involved many complexities beyond the scope of this paper, including more than 2 years of data collection at a worldwide scale. Due to this complexity and scope, we focus here on the real-world knowledge acquisition aspect of the work, and present a few simplified experiments that demonstrate how the acquired knowledge can be used for KBC. The contributions of this paper are primarily:

- To demonstrate that class-level bipartite knowledge acquisition can be effective in approximating instance-level knowledge (Section 5.5) as a solution to *label sparsity*;
- A crowdsourcing approach to acquire such class-level knowledge for the local shopping problem (Section 5.4);
- Experimental results that show the effective combination of class- and instance- level knowledge from various sources used in the launched system (Section 6.3).

The approach has generalized to other bipartite relations between places and types of entities that are organized in a taxonomy, such as dishes at restaurants, services at professional offices, etc., as well as a wide range of other bipartite graph problems where common sense or categorical knowledge prevails as defaults, such as ingredients for dishes, linnean taxonomies of living creatures, etc.

## 2. Formalization

We start with an initial knowledge graph $G^{\prime }(I_{P}\cup \;C,R_{T}\cup \;R_{SC})$, where $C=C_{P}\cup C_{O}$ forms the set of all categories, partitioned into place $\left\{c_{p}\in C_{P}\right\}$ and offering $\left\{c_{o}\in C_{O}\right\}$ categories (e.g., hardware-store, power-tools, resp.), and $\left\{i_{p}\in I_{P}\right\}$ the set of all place instances (i.e., the establishments such as stores and restaurants themselves). The edges of the graph are the class/instance (also known as type) relation between place instances and place categories $\left\{\langle i_{p},c_{p}\rangle \in R_{T}\right\}$, and the subclass relation $\left\{\langle c_{p},c_{p}^{\prime }\rangle \in R_{SC}\right\}$ with a disjointness constraint


$$\langle x,y\rangle \in R_{SC}\Rightarrow \{x,y\}\subset C_{P}⊕\{x,y\}\subset C_{O}$$

so that the relation is only defined over pairs of categories belonging to the same type. Lastly each of these primitive sets are disjoint $I_{P}\cap C_{P}=I_{P}\cap C_{O}=C_{P}\cap C_{O}=\emptyset$, making $G^{\prime }$ tripartite. As usual, $R_{SC}$ forms a partial order within each (place and offering) category partition, and is transitive over the subcategory relation so that $\langle x,y\rangle \in R_{T}\wedge \langle y,z\rangle \in R_{SC}\to \langle x,z\rangle \in R_{T}$. This is meant to capture a traditional kind of knowledge-graph scenario.

**Problem 1**. The *local offerings problem* is the extension of $G^{\prime }$ to $G(I_{P}\cup C,R_{T}\cup R_{SC}\cup R_{I}\cup R_{C})$ through the addition of the *class-level* offering availability relation $\left\{\langle c_{p},c_{o}\rangle \in R_{C}\right\}$ and the *instance-level* offering availability relation $\left\{\langle i_{p},c_{o}\rangle \in R_{I}\right\}$.

**Figure 2:**
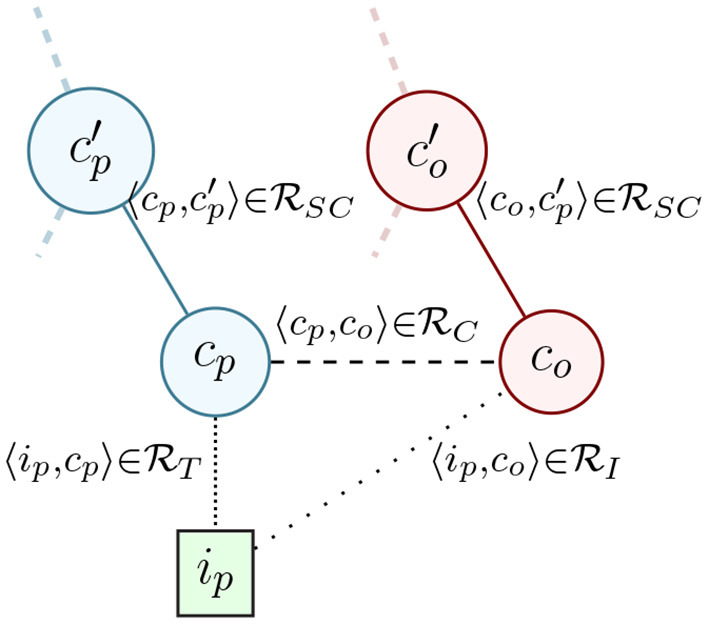
Example subset of graph G with a place instance *i_p_*, a place category *c_p_*, its parent category $c_{p}^{\prime }$, a offering category *c_o_*, its parent $c_{o}^{\prime }$ and the class- and instance- level offering availability relations between them.

The place instances $\left\{i_{p}\in I_{P}\right\}$ represent individual physical places like *Trader Joe's at 142 14th St*. (TJ142), each of which is typed with some number of place categories $\left\{c_{p}\in C_{P}\right\}$ like *Supermarket*. The offering categories $\left\{c_{o}\in C_{O}\right\}$ represent the types of offerings at all places, such as *Milk* or *Dairy*, so that $\left\{\langle TJ142,Milk\rangle \in R_{I}\right\}$ means that particular Trader Joe's sells Milk. Note that a more complete definition of the local shopping problem would include the extension of $C_{O}$ to instances (i.e., place inventory), but we do not have access to that data, and use this definition as a simplification that serves to answer most *local offering* queries. A simple example is shown in Figure 2, showing four categorical graph nodes and one instance node, with each of the relation types shown as edges.

This simplification is best understood as a matrix $\text{R}:I_{P}\times C_{O}$ representing $R_{I}$, where **R**_*i,j*_ are observations (or predictions) that place *i* offers *j*. With enough observed **R**_*i,j*_, collaborative filtering methods (e.g., matrix factorization) can be exploited to predict unobserved values from observed ones. Moving between matrix and graph representation can be done in a variety of ways, such as thresholding matrix values into discrete edges in $R_{I}$, or using a graph formalism that supports confidence values on edges, as described in Noy et al. (2006).

We argue that the real world grounding of the $R_{I}$ association in people's everyday experience allows us to exploit meaningful common sense *categorical* knowledge for the problem of acquiring the edges in $R_{C}$, and use simple defeasable methods to then infer the edges in the graph for the relation $R_{I}$.

## 3. Vocabulary

The local offerings system and all the experiments described in this paper use the open *Google My Business* (GMB) categories^3^^,^^4^ for place categories ($C_{P}$) and *Google Product Taxonomy*^5^^,^^6^ for the offering categories ($C_{O}$). Each set comes with a taxonomic structure that we encode as the $R_{\mathrm{SC}}$ relation, every category has at least one parent category with the exception of the top-level (most general) categories, and a few categories have multiple parents.

This project began with shopping and was extended to dining by adding a number of dishes to $C_{O}$. These dishes are from Google's KG, and most of them can be found in Freebase under the type /food/dish. The restaurant categories are already part of the GMB set.

There are roughly 15k products categories in $C_{O}$, that are similar in semantics to UPCs (Universal Product Code, the bar codes on most packaged products), grounding out in 19 top-level categories. There are roughly 10k dishes in $C_{O}$, that are similar to menu items, with very little taxonomic structure. The GMB categories that comprise in $C_{P}$ include many that are unrelated to local shopping or dining, so we restrict ($C_{O}$) to those below *store* and *restaurant*, resulting in roughly 3k with those two roots.

These taxonomies have different graphical structure: the product taxonomy is fairly deep, and the place taxonomy is fairly shallow, yet they align surprisingly well. For example, there is a deep taxonomy of products under “Grocery,” and a store category “Grocery Store.” There are a few misalignments, for example “Batteries” are under “Electronics” but are sold at “Drugstores.” A few of these misalignments are ameliorated by hybrid categories like “Household products,” which is an additional ancestor for “Batteries.” The food taxonomy we used from Freebase is nearly flat, making for an interesting comparison on the usefulness of a good taxonomy. Note that we do not change the taxonomies or memberships; as defined in Section 2, we treat the initial graph $G^{\prime }$ as given.

Finally, Google Maps has tens of millions of establishments worldwide that form the set of places $I_{P}$; each has a category label which is displayed in the maps UI under the place name and user rating, giving us the edges in $R_{T}$. A large part of these labels are assigned by merchants, some by users, some by operators and others by machine automation. These labels are generally high quality, with precision over 0.8. The largest source of inaccuracies are store labels that are more general than they need to be, when a more appropriate category exists. The labeling infrastructure requires a single “primary” category, while many places could be categorized in several ways. A Glossary of terms defined in this paper has been provided in Table 1.

**Table 1:** Glossary of terms.

**Terms**	
Place	An establishment (store or restaurant) on Google Maps
Offering	A product or dish available at a place
KBC	Knowledge Base Completion
GMB	Google my Business (source store categories)
GPT	Google Product Taxonomy (source product categories)
UGC	User Generated Content–user responses to yes/no questions
CS	Crowd Sense, our approach
WebIE	Information extraction of offering names from place web pages
WALS	Matrix factorization using WALS to predict 〈*i_p_, c_o_*〉 pairs
**Knowledge graph**	
$\left\{i_{p}\in I_{P}\right\}$	Set of place instances
$\left\{c_{p}\in C_{P}\right\}$	Set of place categories
$\left\{c_{o}\in C_{O}\right\}$	Set of offering categories
$\langle c_{p},c_{p}^{\prime }\rangle \in R_{SC}$	Place subclass/superclass relation
$\langle c_{o},c_{o}^{\prime }\rangle \in R_{SC}$	Offering subclass/superclass relation
$\langle i_{p},c_{p}\rangle \in R_{T}$	Place instance/class type relation
$\langle c_{p},c_{o}\rangle \in R_{C}$	Class-level offering @ place availability relation
$\langle i_{p},c_{o}\rangle \in R_{I}$	Instance-level offering @ place availability relation
$G^{\prime }$	Base KG of place/offering classes and place instances
G	$G^{\prime }$ extended with $R_{C}$ and $R_{I}$
R_*i,j*_	Likelihood that place instance *i* sells offering class *j*
**Crowd task**	
*w*_*x,o*_	Rater score for place (class or instance) x and offering class o
α_*c,o*_	Number of “always” answers for class-level pair 〈*c, o*〉
ν_*c,o*_	Number of “never answers for class-level pair 〈*c, o*〉
*y*_*i,o*_	Number of “yes” answers for instance-level pair 〈*i, o*〉
*n*_*i,o*_	Number of “no” answers for instance-level pair 〈*i, o*〉

## 4. A Three-Tiered crowd

The system for which we performed the crowdsourcing described in this paper is quite large and complex, and is launched and available to users worldwide through search. It uses a DNN model to predict $R_{I}$ pairs from many signals that include information extraction (IE) from store web pages, direct merchant feeds, store type, and dozens of other features that include a significant amount of user-generated content (UGC).

The well-known bipartite problems that have been solved by machine learning have the advantage that the organizations that solved them had a lot of labeled data for those problems. For example, Netflix has millions of 〈user, movie〉 pairs, and can use this massive data to seed big machine learning systems to better predict what movies a user make like. A vast number of practical bipartite problems, however, have very little data, resulting in *label sparsity*.

**Figure 3:**
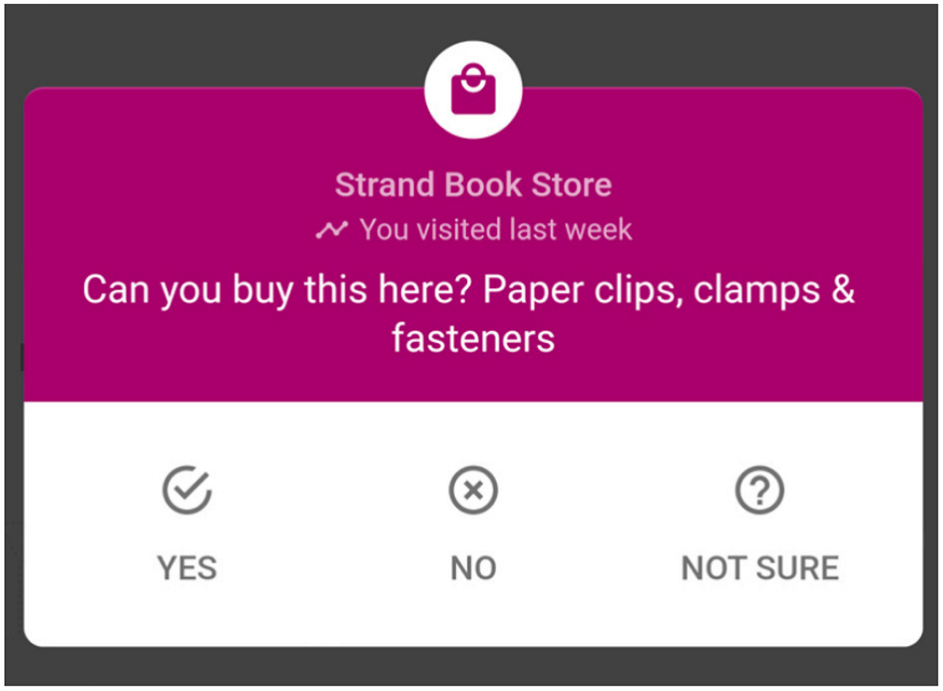
Example question used to gather UGC.

Label sparsity means that machine learning systems don't have enough data to make reasonable predictions, and the only way to move forward is to acquire it. Acquiring the data needed to seed large scale AI systems is as much a problem today as it was during the bygone era of expert systems, where, according to Shortliffe and Buchanan (1975) and many others, the bulk of the research focus was on algorithmic solutions to rule-based reasoning problems, but the bulk of the difficulty and work was in knoweldge acquisition. This history continues to repeat itself; Sambasivan et al. (2021) point out that knowledge acquisition is viewed as less glamorous than inventing new neural algorithms and architectures. As noted above, for the local shopping and dining problems, existing sources gave us less than 0.001% of the total matrix **R**, leaving a huge knowledge acquisition problem. We developed a novel three-tiered crowd to gather the data discussed in this paper:

- **CrowdSense** (CS): We collected 25k class-level $\langle c_{p},c_{o}\rangle \in R_{C}$ pairs for shopping and 20k for dining, from a pool of paid raters. Though a relatively small crowd effort, this ends up being the largest source of instance-level $\langle i_{p},c_{o}\rangle \in R_{I}$ pairs through default inference (full details in Section 5), yielding billions of instance-level pairs.
- **UGC**: Google Maps provides the facility for users to voluntarily add reviews, photos, venue categorization, and attributes (e.g., “has Wi-Fi”) to places they've visited. Through the UGC framework, users answer yes/no questions about product and dish availability at places they've visited, shown in Figure 3. While Google's deployed local search system does use all the UGC data, including reviews and photos, etc., in this paper we only describe and analyze the impact of the yes/no questions, which comprise the largest crowdsourcing element of the system, at millions of answers per day. Each user is given a set of $\langle i_{p},c_{o}\rangle$ pairs to answer, giving us a distribution of yes and no answers for each pair. In the experiments shown in Section 6, we show the growth in coverage over time as more answers are collected, yielding hundreds of millions of instance-level pairs over the course of this study (2 years for shopping and 15 months for dining).^7^
- **Gold**: We collected 40k gold standard $\langle i_{p},c_{o}\rangle$ pairs for shopping, and 20k for dining, by having paid operators call each place *i_p_* and ask them if they sold or served *c_o_*. The places were selected from among more than 50 countries with the top-5 countries being US (20%), JP (5%), IN (5%), GB (5%), BR (4%); places within each country were sampled uniformly to provide a microcosm of representative demographics. Clearly the highest fidelity and most expensive data, it is by far the smallest.

One of the critical obstacles to gathering this data from people in all tiers is the class imbalance: less than 4% of the possible store-offering pairs are positive. Gathering 96% negative results is a waste of human labeling resources and, far more critical, makes for an unsatisfactory user experience—users want to feel helpful and answering 9/10 negative questions is frustrating. Moreover, particularly obvious negative questions, like fish heads at a hardware store, confuse some users into saying they are unsure—the questions are so obvious they feel they must be missing something. Finally, a few of these obvious negatives end up on social media as jokes, which is embarassing.

Active learning (AL) is a known method for dealing with class imbalance—sampling near the classifier boundary typically yields a good balance between positives and negatives and, thus, provides utility for training the model. Unfortunately for problems where there is also label sparsity, there is not enough data to train a model and so nothing to base AL on. Our class-level approach offers a solution to this problem as well. As described in more detail in Section 5, we gather a distribution of judgements on class-level pairs, and the resuling pairs fall into three categories: *obviously available* (e.g., grocery store, milk), *obviously unavailable* (e.g., hardware store, fish heads), and *possible* (e.g., hardware store, 9 inch nails). The *possible* category of class-level pairs captures products that are available at some, but not necessarily all, stores in the class, and provide excellent guidance for selecting instance-level pairs to ask users.

Even after we'd acquired enough data to begin training a model and use AL, the *possible* category offered an additional benefit. In the early stages of acquiring training data, known as the explore (vs. exploit) stage, $\langle i_{p},c_{o}\rangle$ pairs with enough evidence to be close to the classifier boundary are very likely to be positive, so much so that the class balance of margin sampling was 80% positive. Clearly a 50% class balance could then be achieved by up-sampling pairs that are further below the classifier boundary, however such an approach is very likely to choose these problematic obvious negatives discussed above. A mix of possibles with margin sampling was able to achieve a 50% class balance with high utility and no embarassment.

For the Gold data, class imbalance presents as much of a problem as for UGC, however since this data set is used to measure the quality of the CS data, we did not want to bias our evaluations by using CS as a guide. Instead, to achieve better class balance, the WebIE baseline data (q.v. below) was used to guide the collection toward pairs that had an increased chance of being true; for example, if a places's webpage mentioned an offering we would try to call places of the same type and ask about that offering. We enforced a positive/negative class balance of 50%, and targeted a stratification of the sampling that preserved the 30/70 balance of places with and without websites.

## 5. Crowd Sense

The obvious way to gather the edges in $R_{I}$ would be to use store inventory or transaction records. The problem with this approach is that *local offerings* is still mostly an off-line or highly siloed process worldwide, and we did not have access to transactional data that gives us these observations. Google provides merchants a free way to share their menus or inventory on-line, but much fewer than 1% of places worldwide had made use of it. Our data showed that web pages and merchant feeds together accounted for less than 0.001% of the space of the matrix **R**, giving us the label sparsity problem. Filling the cells of matrix **R** means acquiring the edges in $R_{I}$, and we propose to accomplish this by starting with the acquisition of edges in $R_{C}$, the *class level attributes*, and inferring those values as defaults for $R_{I}$.

### 5.1. Crowd Hypothesis

The intuition driving our approach is that the crowd can provide the class-level knowledge ($R_{C}$) by appealing to their common sense experience; everybody knows that, e.g., “All supermarkets sell milk.” Reality is more complicated, and since the problem space is sparse, the class-level data is also dominated by what offerings are obviously *not* available. Far behind the obvious negatives are, as discussed above, the possibles—offerings that are *usually*, but not always, available at some type of establishment. Wasabi Peas, while they are found almost exclusively in grocery stores, are not found in all of them. What we really aim for the crowd to provide is a *distribution* of the offerings available at places of a given type. This is where a lot of existing knowledge graph methods fail, especially at the class-level, as they rely on an assumption of discreteness.

It may seem that we could ask individual people to answer a question like, “What percent of stores of type *c_p_* sell product *c_o_*?” However, research in human computation such as Surowiecki (2005) has shown that individuals cannot reliably answer such questions. Using (Welty et al., 2012; Aroyo and Welty, 2014, 2015) as a starting point, we hypothesized:

**Hypothesis 1**. Asking multiple raters about the same categorical pairs would produce a distribution of answers that approximate the real world distribution of $R_{I}$.

In other words, if 70% of raters say that oat milk is sold at grocery stores, then 70% of grocery stores will sell oat milk.

Before testing our hypothesis, we ran numerous pilots to tune the hyper-parameters of the crowd task in the shopping domain, asking raters questions about 11k $\langle c_{p},c_{o}\rangle$ pairs from 154 store types and 3600 products in five countries. We experimented with: the number of raters per pair, testing between 5 and 25 raters per pair; the size of the rater pool, ranging from 100 to 500; the question phrasing; and the answer options. Based on manual analysis of the cost and quality, we settled on these task hyper-parameters: five raters per pair, randomly selected from a pool of 130 raters in six countries, sourced from contracted operators through an in-house crowdsourcing platform, and the question, “Would you expect to find *c_o_* products in stores of the category *c_p_*?” with four answer options (“Always Available,” “Sometimes Available,” “Never Available,” “I don't know”). For dishes, the question was rephrased, “Would you expect to find *c_o_* dishes in restaurants in the category *c_p_*?”

Under these settings, our final PRODCAT task (see below) gathered 25k class-level ($\langle c_{p},c_{o}\rangle$) pairs with 5 labels per country, that through inference (*q.v*. Section 6.1) resulted in billions of $\langle i_{p},c_{o}\rangle$ pairs, 99% of which were negative. It took 6 weeks to run and analyze the pilots, and 2 weeks to run the final task. For dishes, the MATRIX task collected 15k class level pairs from 5 raters per pair in 2 weeks, resulting in billions of instance-level pairs.

Raters were supplied by a set of contractors who are obligated to follow Google's Code of Conduct, and were managed by an administrator outside our group. The MATRIX and PRODCAT task designs (q.v. below) grouped between 200 and 400 pairs in a single matrix, raters were assigned a matrix by the administrator based primarily on availability. Many raters were assigned multiple matrices over time, but in our analysis we did not account for individual characteristics of raters (such as expertise), even though we know from Aroyo and Welty (2014) this can yield improvements.

### 5.2. Data Collection Tasks

Another way to state our hypothesis is that the categorical crowd disagreement should reflect the real world distribution, but disagreement can have many causes that are not related to the desired distribution. The various pilot tasks we ran represented a gradual refinement of the data and task descriptions to eliminate disagreement from other causes. We report here on four different approaches for the shopping domain:

#### 5.2.1. RANDOM

To confirm the sparsity of $R_{C}$, we randomly and independently selected category pairs from $C_{P}\times C_{O}$, weighing the selection from $C_{P}$ proportionally to the number of stores belonging to each category (i.e., larger categories are more likely to be selected). Pairs were presented to 5 raters from the same country. This RANDOM task confirmed that the vast majority of pairs are “obvious” negatives (asphalt at grocery stores, cars at violin shops, etc.), as more than 95% of the pairs resulted in 5 “Never” ratings.

#### 5.2.2. SINGLETON

To address the sparsity shown in RANDOM, we leveraged web signals (see Section 6.1) to select pairs with more likelihood to be available at places within a given category, and presented one pair at a time to 5 raters from the same country. This resulted in a distribution of rating scores ranging from all-5 “Always Available” to all-5 “Never Available” skewing toward the positive (always) side. The SINGLETON task results showed disagreement from other causes, described in Section 5.3.

#### 5.2.3. MATRIX

**Figure 4:**
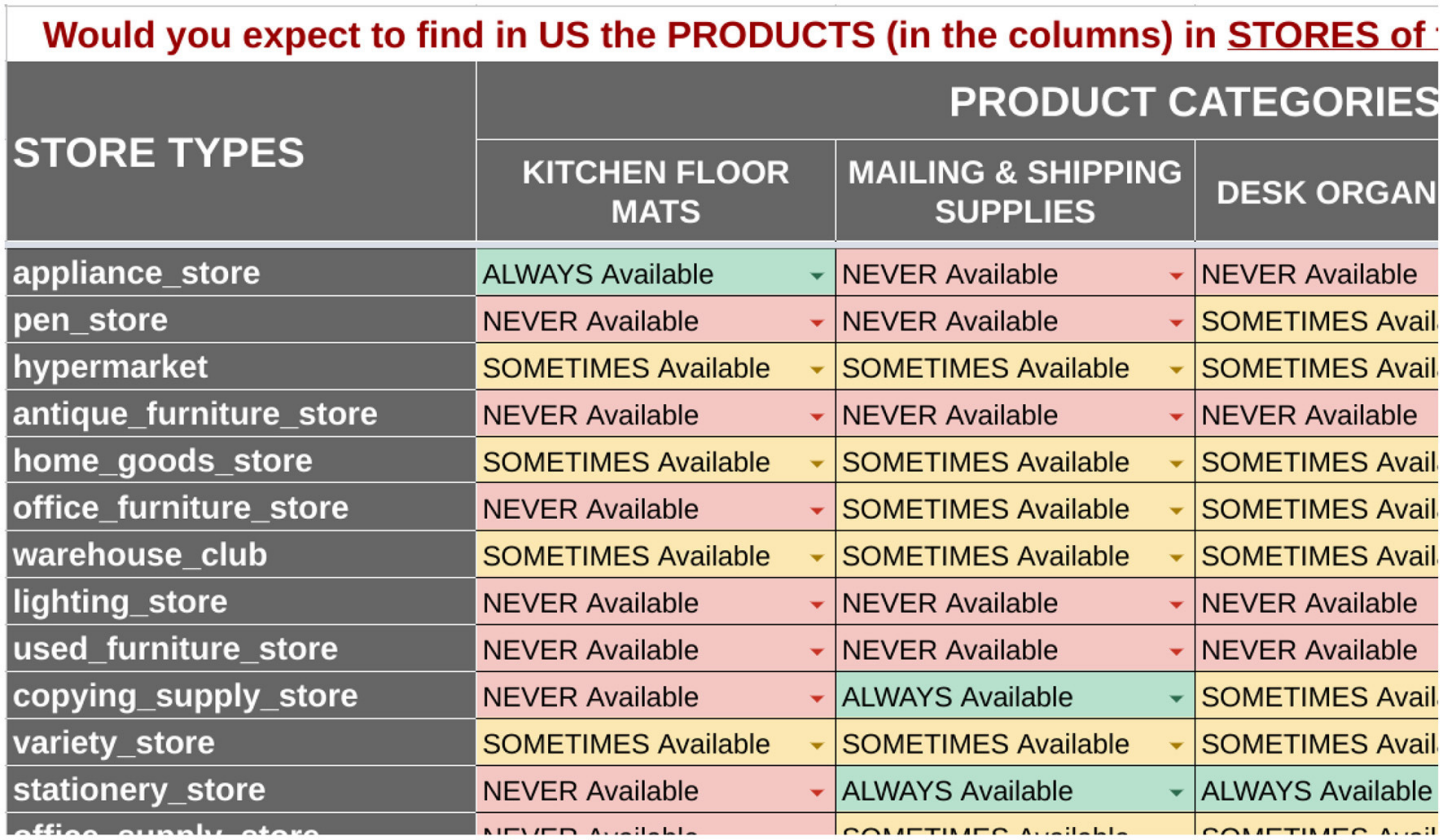
Partial view of the PRODCAT data collection template with example answers from one rater.

To address the disagreement due to ambiguity (Section 5.3), we designed a novel matrix presentation of class-level pairs, with four $\left\{c_{o}\in C_{O}\right\}$ as the columns and a set of 100–200 $\left\{c_{p}\in C_{P}\right\}$ as the rows, depending on our ability to match offerings to the place categories using web signals. Figure 4 shows the matrix presentation (with data sampled through the PRODCAT method below). The advantage of this presentation is that raters familiarized themselves with a category and answered many questions related to it, rather than having to understand one pair at a time. This approach still produced some unwanted disagreements due to difficulty understanding some of the products, esp. very specific ones, and we were concerned that the web signals were biasing our sample toward availability patterns of online places, rather than our target class of establishments without web pages. Most importantly, the amount of time the raters spent per $\langle c_{p},c_{o}\rangle$ dropped by 50%.

#### 5.2.4. PRODCAT

The final crowdsourcing task used the MATRIX presentation but changed to a dynamic method that sampled the $\langle c_{p},c_{o}\rangle$ pairs starting at the top of the product taxonomy, and working down the $R_{\mathrm{SC}}$ relation from most general to most specific. It was not useful to treat the store taxonomy this way, as it is very shallow, and we did not have a dish taxonomy. When a pair was given an overall negative label, we did not sample any subcategories of *c_o_* and inferred a negative label for all descendents. For example, since *Auto parts stores* do not sell *Grocery* and $\langle Dairy,Grocery\rangle \in R_{SC}$, we did not ask 〈*Auto parts stores, Dairy*〉.

The product taxonomy is not a strict tree, but a DAG, and when reconciling conflicting ratings from multiple parents, we retained the most positive rating. *Electronics* are not sold at *Pharmacies*, whereas *HouseholdProducts* are sometimes sold there, and *Batteries* are a subcategory of both *Electronics* and *HouseholdProducts*, so we do ask about 〈*Batteries, Pharmacies*〉.

This top-down taxonomic pruning eliminated any need for the web signals, and accounted for the sparsity at a very high level, since (by accident or ontology) the store and product categories were well aligned: e.g., *Auto parts stores* sell *Auto parts* and do not sell *Groceries*. Higher level categories also made a lot more sense to raters when presented with a sub-category, e.g., *Sports and Outdoor Electronics* with *Fitness Trackers*, and since our rater pool did not vary much, they became familiar with the taxonomic distinctions as they progressed down the taxonomy, which was evidenced by a reduction in visits to the taxonomy element descriptions over time.

#### 5.2.5. Dish MATRIX

To gather the class-level pairs $\langle c_{p},c_{o}\rangle$ for the dining domain, we were not able to fully reuse the PRODCAT method, since the dishes in our KG did not have taxonomic organization, which was the key to the improvements of PRODCAT over MATRIX. Instead, we used the MATRIX method, presenting the class-level pairs in a matrix, selected by their popularity in web signals. As with singleton, this approach favored positive pairs, indeed our raters appear to have been overly positive in their answers.

### 5.3. Ambiguity

In the pilot experiments run for shopping we observed disagreement in the results that did not support our crowd hypothesis, but were caused by ambiguity such as:

- product is a material, substance (e.g., plastic, starch, arugula) or some product aspect (e.g., color, size)
- product is a brand (e.g., Avian, Kleenex) or contains a brand name (e.g., Nike Sneakers, Todd's boots)
- place or offering is too specific (e.g., duck sauce, goat meat, vanilla orchids, banner store)
- place or offering is too generic (e.g., gift, organic food, chicken, restaurant)
- offering is regional (e.g., Harissa, Jajangmyeon)
- offering is seasonal (e.g., christmas trees, flip-flops)
- offering is polysemous in a way that is resolved by the store type, e.g., “fish” in a grocery store vs. a pet store
- flashy menu item (e.g., nacho fries bellgrande, del monde delux).

In MATRIX and SINGLETON, for example, raters seem more willing and able to answer the question, “Is milk sold here?” compared to “Is dairy sold here?” In the latter case, there is uncertainty over what minimum set of dairy items (milk, cheese, butter, yogurt, etc.) would be needed for “sells dairy” to be true, yet the equally rich sub-categories of milk (whole milk, skim milk, organic milk, etc.) did not cause the same uncertainty. When presented with the categories in a top-down fashion, raters first dealt with their uncertainty about “dairy” and applied it to the subcategories as well, and this handled most of the general and specific ambiguity, and for many store types, raters were willing to give definite answers about the other sub-types in subsequent tasks.

We specifically addressed the material, aspect and brand problems by removing them from the product set, their treatment is the subject of future work. We instructed the raters to treat seasonal products as “year round,” after confirming that users are less likely to search for such products out of season. We updated the task design to allow raters to explore the two taxonomies to help with polysemy, but we found that grouping store categories by taxonomic (sibling and parent) relations in PRODCAT obviated this exploration.

Regional products produced disagreement esp. across countries, where for the final tasks we sourced raters in six countries (US, IN, BR, FR, JP, IN). Often this showed up merely as “I Don't Know” answers which were not used in predicting $R_{I}$, but do show up in IRR. More interesting cases included when a product had a slightly different meaning, or was sold in different types of stores, in different regions. For example, “syrup” in France is sold in drug stores, and raters in other countries did not agree. This is because in France “syrup” is cough syrup, and this association did not exist elsewhere that we tested. We had many expectations for the role of, and differences between, raters in different countries, described in more detail in Section 5.6. Despite these anectdotal examples, class-level ratings from one country were generally worse at predicting instance-level availability within the same country, and better at predicting other countries. In the final system, we ignored the country of the class-level ratings, treating all raters as equal.

Flashy menu items, in which superlatives and other postive-sentiment modifiers are added to dish names, were an additional problem in dining that we did not observe in the shopping domain. This is in part due to the taxonomy curation of the shopping data, in which such modifiers had been removed to create a fairly neutral set of categories. For dining, which lacked the taxonomy, some raters were able to identify the superlatives as meaningless, or were familiar with the dish names because they came from well-known chains, while other raters didn't have that knowledge and would answer either negatively or uncertainly. Our scoring method effectively neutralizes such dish names (see Section 5.5), as the disagreement moves the score close to zero, and we did not choose to address it otherwise. Our current work seeks to address this problem through the automatic development of a taxonomy.

### 5.4. PRODCAT Data Collection Task

The final design of the PRODCAT task, which was only used in the shopping domain, presented a matrix of $\langle c_{p},c_{o}\rangle$ pairs to raters in six countries, five raters per country, and consisted of several elements:

- a list of store categories, $c_{p}\in C_{P}$
- a list of product categories, $c_{o}\in C_{O}$
- *c_p_, c_o_* pairs presented in an *n* × 4 matrix, where each *c_p_* is a row and each *c_o_* is a column; *n* ranged from 40 to 200 depending on our ability to find suitable products
- the matrix was prefaced with: “Would you expect to find in *country* the products (in the columns) in stores of the types (in the rows)?”
- each cell in the matrix connected one pair with four possible answers: “Always available,” “Sometimes available,” “Never available,” and “I Don't Know”
- the row and column headers *c_o_* and *c_p_* included links to an image, a short description, and the position in the respective taxonomy
- raters were encouraged to explore the taxonomies in order to better understand categories
- The column product types were chosen such that three were taxonomy-related (sibling or more-specific child) and one was not, e.g., “aspirin,” “notebooks,” “paper supplies,” and “lined paper.”

The final matrix PRODCAT crowd template is shown in Figure 4 with an example of answers provided by one rater. Based on rater feedback and metrics shown in Section 5.5, this presentation helped resolve many forms of polysemy mentioned in Section 5.3.

### 5.5. Error of Class-Level Ratings

Table 2 shows a small sample of the CS task results for $R_{C}$ pairs; we have intentionally downsampled the “5-never” pairs to show a mixture of different vote ratios.

**Table 2:** Example CrowdSense ratings on $R_{C}$ pairs.

**Category**	**Product**	**Always**	**Some**	**Never**
Auto parts store	Pita	0	0	5
Bakery	Longline Vests	0	0	5
Beauty supply store	Aromatherapy	5	0	0
Bicycle store	Home furnishings	0	0	5
Butcher shop	Quicklime	0	0	5
Chinaware store	Watches	0	0	5
Clothing store	Women's shirts	5	0	0
Clothing store	Petite negligee	5	0	0
Clothing store	Truck tailgate caps	0	0	5
Clothing store	Chameleon	0	0	5
Clothing store	Typewriter ribbon	0	0	5
Coffee store	Instant coffee	4	0	1
Cosmetics store	Non-dairy milk	0	0	5
Drugstore	tarragon	0	0	5
Electronics store	Canister vacuums	5	0	0
Feed store	cybex	0	0	5
Fresh food market	Work dresses	0	0	5
Fruits and vegetables	Turkey sausage	0	1	4
Furniture store	Canopy beds	4	1	0
Furniture store	Box springs	4	0	1
Grocery store	Smart light bulbs	0	0	5
Grocery store	Frozen clams	5	0	0
Grocery store	Soy nuts	4	1	0
Home goods store	Storage baskets	4	1	0

In Welty et al. (2021) we showed that inter-rater reliability (IRR) cannot reflect the quality of ratings where disagreement is the desired result, so we report the *error* of different $R_{C}$ pairs in predicting the distribution of $R_{I}$ pairs, by comparing ratings-based scores on $R_{C}$ pairs against UGC scores on $R_{I}$ pairs obtained from users (see Section 4). Each class and instance level pair has a score:


$$w_{x,o}=\{\begin{array}{ll} \left(\alpha_{x,o}+\frac{1}{2}\sigma_{x,o})/(\alpha_{x,o}+\nu_{x,o}+\sigma_{x,o}\right) & \text{if }x\in \;C_{p}\\ y_{x,o}/(y_{x,o}+n_{x,o}) & \text{if }x\in I_{p}\; \end{array}$$

where α_*x,o*_ is the number of “always” answers for class-level pairs 〈*x, o*〉, σ_*x,o*_ the number of “sometimes,” and ν_*x,o*_ the number of “never” answers; and *y*_*x,o*_ is the number of “yes” answers for store instance-level pairs 〈*x, o*〉 and *n*_*x,o*_ the number of “no” answers.

Next let $I_{c}=\{i:\langle i,c\rangle \in R_{T}\}$ be the instances of place category *c* under *R*_*T*_. The mean absolute error of class-level pair 〈*c, o*〉 is:


$$\text{MAE}(\langle c,o\rangle \in R_{c})=\frac{\sum_{i\in {ℐ}_{c}}\left|w_{i,o}-w_{c,o}\right|}{\left|{ℐ}_{c}\right|}$$

**Figure 5:**
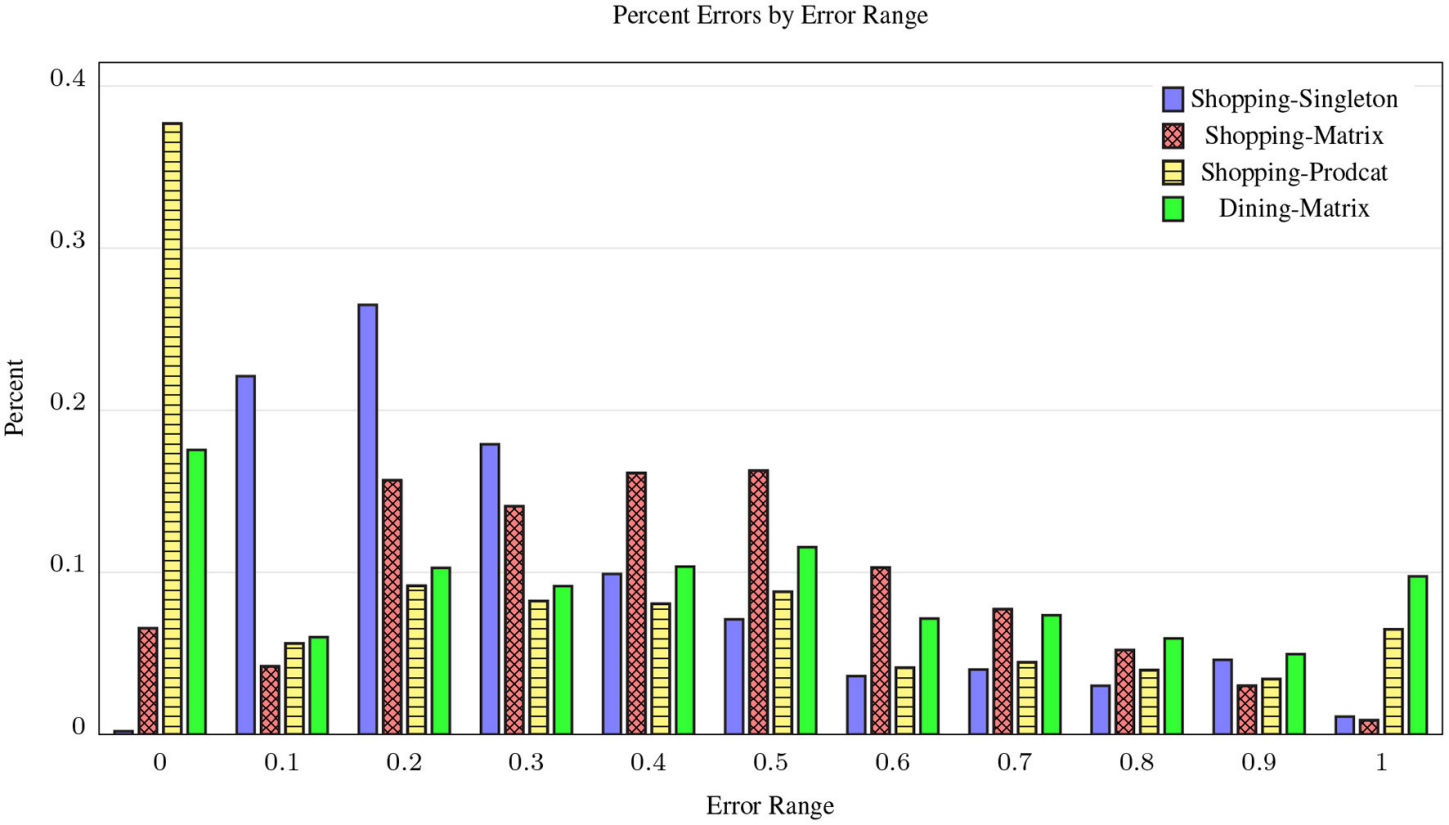
Histogram of Normalized-MAE on CrowdSense pairs for three shopping and one dining (Section 5.5) class-level crowd task designs. Bins to the left indicate the relative number of pairs with lower error, making Shopping-PRODCAT the clear leader. Dining-MATRIX performs better than shopping MATRIX.

The idea is that if the class-level scores (*w*_*c,o*_) are an accurate prediction of the availability distribution at the instance level, then they should model user observations at individual stores (*w*_*i,o*_), averaged over the size of the store category ($\left|I_{c}\right|$). Figure 5 shows the distribution of MAE scores per category pairs for the three shopping and one dining data collection tasks. Despite PRODCAT being a harder task for raters due to the sampled pairs, it performs much better than the other shopping tasks, with nearly half of its categories scoring in the lowest error range, clearly supporting our crowd hypothesis: the disagreement on $\langle c_{p},c_{o}\rangle$ pairs approximates the distribution of $\langle i_{p},c_{o}\rangle$ when $\langle i_{p},c_{p}\rangle \in R_{T}$, according to user observations. For Dining, we only ran the MATRIX task, to replicate as much as possible the results from Shopping. As expected, the MAE is lower than for PRODCAT on shopping, but considerably better than MATRIX for shopping. One explanation for this is that our raters were more familiar with dining around the world than shopping, and there was less disagreement caused by not understanding the pair.

### 5.6. Error of International Ratings

Another hypothesis we formed early on was that raters in our class-level rating pool, which was international, would know their own countries better than other countries, and the initial design of the system called for increasing the weight of in-country class-level ratings over out-of-country ratings when calculating *w*_*x,o*_ (see above). In our analysis of CrowdSense errors in the pilot studies, we certainly saw examples of raters misunderstanding dishes and products from other countries (see Section 5.3).

**Figure 6:**
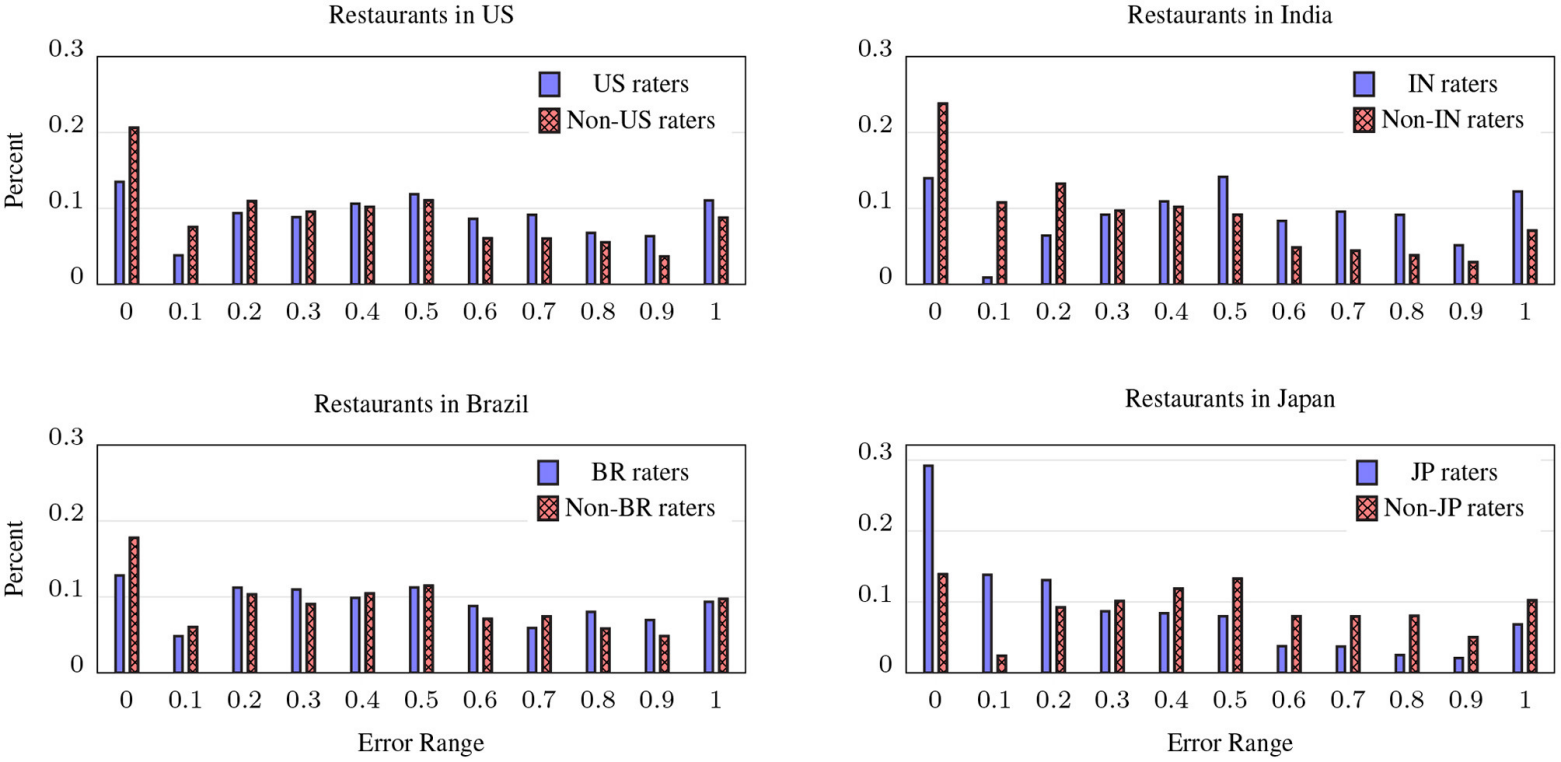
Distribution of CrowdSense errors (Normalized-MAE) for ratings in four countries, comparing CrowdSense predictions from raters in each country to raters outside that country. A shift of scores to the left indicates lower overall error; surprisingly, for all countries except Japan, out-of-country CrowdSense raters are more accurate than those within the country.

This hypothesis was mostly supported by our analysis of the shopping data, but it turned out to be largely false for dining, to our great surprise, as shown in Figure 6; as with Figure 5, the charts show the distribution of the normalized MAE from CrowdSense predictions, but in each chart we've restricted the actual restaurants to those within the indicated country, and calculated the *w*_*x,o*_ scores for CrowdSense for raters in the country (solid blue bars) and for raters not in the country (hashed red bars). With the exception of Japan, outside raters have a lower error rate, as their distributions are shifted significantly to the left. In Brazil, the effect is small, in the US it is large and in India the largest. In Japan, the expected effect is dramatic—Japanese CrowdSense raters were far better at predicting the distribution of dishes at Japanese restaurants than non-Japanese raters. We ran the experiment for Germany and Indonesia (not shown) with similar results as the US and India.

For the US, this may be explained by the fact that there are far more chain restaurants that dominate the numbers when calculating the MAE, and many of these chains are familiar abroad, so while US raters are making their decisions based on a broader perspective of chains and non-chains, non-US raters are making their decisions based only on chains, and these capture a larger piece of the US restaurant landscape. In addition, the US has far more restaurants serving international cuisines than any other country, making it possible for international raters to know something about more US restaurants. For Japan, more than any other country, there are many restaurants that serve only a very specific kind of food, and this is well known in Japan and not as much outside it. A possible explanation for the counter-intuitive results in the other countries is that the restaurant taxonomy does not cover those regions very well, leaving more restaurants mis-categorized.

## 6. Instance-Level Prediction Experiments

### 6.1. Data Sources

We compare and contrast several approaches for acquiring and predicting the relations in $R_{I}$:

**CrowdSense** (CS): Class-level associations $\langle c_{p},c_{o}\rangle \in R_{C}$ and an associated score for each pair *w*_*c_p_,c_o_*_, collected through PRODCAT (as described above) for shopping, and MATRIX for dining. In our experiments, we treated the CS data as a static set, although in practice it could grow or change over time like UGC. We collected 25k pairs in the shopping domain and 20k for dining.

**User Responses** (UGC): As described in Section 4, we collected more than 100M instance-level pairs for shopping from volunteer users around the world over a 2 year period, and roughly half that amount over a 15-month period for dining. Most of the UGC pairs have a distribution of yes and no answers, and more sophisticated processing of the answers is possible, but for simplicity we use the majority vote as the label in the experiments below, where we break the data into sets representing the first *n* ∈ [1, 24] months of collection, to illustrate the growth of the data over time.

**Figure 7:**
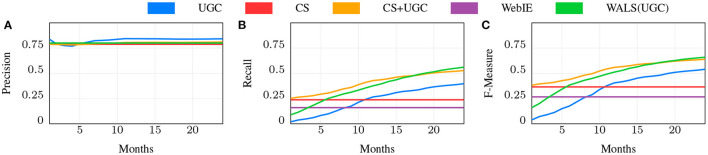
Precision, Recall, and F-measure for different ways of predicting $R_{I}$ for shopping.

**Figure 8:**
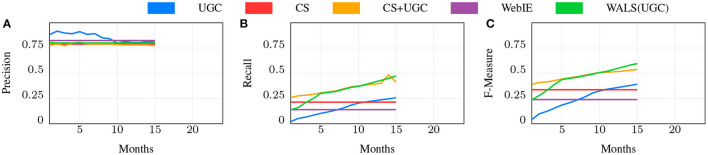
Precision, Recall, and F-measure for different ways of predicting $R_{I}$ for dining.

**Web baseline** (WebIE): The baseline approach to supporting *local* queries is the Web: using product or dish names mentioned on each place's registered web site as part of an inverted index that are matched to search queries for those products. As discussed above, this approach for local shopping is limited by the coverage of local (aka brick and mortar) stores and restaurants on the web, which was under 30% (60% for the US) at the start of this project in 2017, and has not increased substantially in the years hence. We used a named entity recognizer to extract instance-level pairs ($R_{I}:I_{P}\times C_{O}$) for places with a web site that mention offerings on any of the site's pages, and used the extraction confidence probability threshold yielding 80% precision. WebIE is only able to obtain positive labels, leaving negatives to be inferred from the complement. We chose the 80% precision threshold as this is roughly the precision of the CS inferred data (see Figures 7, 8), which we compare to this and other data sources. While other Web sources (user reviews, coupons, photos, search keyword click-throughs, etc.) and more advanced entity extraction techniques such as Wang et al. (2020) might improve the recall, for most places this information simply is not available. We treated the Web as a single unchanging dataset; for our experiments, the change over time was not significant enough to measure.

**WALS(UGC)**: Since predictions of the instance-level pairs form a matrix, **R**, an obvious approach is to use matrix factorization on the matrix formed from data gathered using the above methods. We used an off-the-shelf WALS implementation based on Koren et al. (2009) trained on the UGC scores discussed below. Since WALS does not use “features,” but rather a matrix of real values, we did not include other inputs to WALS in Figure 7 or Figure 8.

### 6.2. Evaluation

Ultimately our goal is to enable offering queries like, “where can i buy a raincoat?” or “where can i get sesame chicken?” to return nearby places on maps as well as (web) search results; however, direct application impact metrics from our system, which launched in mid-2020, are proprietary. Here we focus on the knowledge acquisition part of the system using metrics of knowledge-based completion, see for example (McNamee and Dang, 2009; Welty et al., 2012).

We collected 40k gold standard $\langle i_{p},c_{o}\rangle$ pairs for shopping, and 20k for dining, by having paid operators call each place *i_p_* and ask them if they sold or served *c_o_* (see Section 4). We used these pairs as a test set in the experiments below. When evaluating against the gold standard, any instance-level pairs that are present in the gold set but missing in the evaluated data are counted as false negatives toward recall. Table 3 shows a small sample of the shopping gold standard pairs, and Figures 7, 8 show the results on 24 and 15 months of UGC data, resp. Note that since WebIE was used to guide the collection of the gold standard, it has a slight advantage in the evaluation.

**Table 3:** Example gold standard $R_{I}$ pairs.

**Store**	**Category**	**loc**	**Product**	**Available**
7-Eleven	Convenience store	US	Distilled water	FALSE
ALDI	Grocery store	US	Fruitcake	TRUE
AURORA MKT	Store	US	Men's Gloves	FALSE
Adams Pharmacy	Pharmacy	US	Kool aid	TRUE
Ag construcciones	Building materials	PY	Blinds	TRUE
Alanyurt Gıda	General store	TR	Razor blades	TRUE
Amorino	Ice cream shop	FR	Meat	FALSE
Barnes and Noble	Book store	US	Blankets	FALSE
Barstow Buick	Car dealer	US	Crown victoria	TRUE
Barstow Buick	Car dealer	US	Gears	TRUE
Bazar	bazar	BR	Mary kay	FALSE

### 6.3. Results

#### 6.3.1. WebIE

Since the values on the WebIE data for each $\langle i_{p},c_{o}\rangle \in R_{I}$ are fractional in [0, 1], we determined the lowest threshold with at least 0.80 precision and computed recall based on that, resulting in a recall of 0.136 at 0.80 precision for shopping, and a near-identical 0.139 for dining. This recall reflects the fraction of places with web pages, the fraction of offerings (products or dishes) mentioned on those pages, and the recall of the named entity recognition. We did not independently measure these other factors, as Web performance was merely a baseline. WALS on WebIE data was not able to show very significant improvement, and the results are not shown.

#### 6.3.2. CS

The primary hypothesis of this paper is that the acquisition of class-level associations in $R_{C}$ from the crowd is an effective way of rapidly jump-starting instance-level associations in $R_{I}$. As described in Section 5, we acquired 25k class-level pairs from a paid crowd for shopping and 20k for dining, each with a score *w*_*x,o*_ (see Section 5.5), and chose the following simple procedure to infer the instance level pairs:


$$\begin{array}{l} w_{x,o}>0.5\wedge x\in \Rightarrow \langle x,o\rangle \in R_{c}\\ \langle x,o\rangle \in R_{c}\wedge \langle y,x\rangle \in R_{T}\Rightarrow \langle y,o\rangle \in R_{I} \end{array}$$

In other words, for class level pair 〈*x,o*〉, if *x* is a place category, and the majority of raters (*w*_*x,o*_ > 0.5) answered that you can find *o* at places of that type, add a class-level edge to G, and an instance-level edge to every instance of place category *x*.

We then measured the effectiveness of the CS by comparison of the inferred edges in $R_{I}$ to the Gold set, achieving a recall of 0.238 with a precision of 0.788 for shopping, and 0.214 with a precision of 0.788 for dining. While this shows a distinct improvement over WebIE, of interest is the combination, which improves recall to 0.351—near perfect complementarity—while slightly losing precision at 0.782 (for simplicity we do not show this in Figure 7 or Figure 8). The combination uses the WebIE or CS signal if the other is not present, and the CS signal if they are both present, since the CS data includes negatives and WebIE does not. (WALS inference was ineffective here; see below).

#### 6.3.3. UGC

The UGC dataset grows over time as more users visit places and answer questions, while we treat the Web and CS data as constant (see above). We expect that, given enough time, UGC will overtake CS and WebIE in recall, so an important question is how much time the CS data is worth compared to UGC, and whether it continues to show value. In Figures 7, 8, the blue line shows the precision, recall, and F1 score of the UGC data using the majority vote as the label, and the red line shows the CS performance, which, as noted above, doesn't change. In both shopping and dining, the UGC line crosses the CS line at around 11 months, indicating that CS is worth about 11 months of UGC collection in both domains.

#### 6.3.4. WALS(UGC)

We populated the matrix **R**_*p,o*_ from UGC *w*_*p,o*_ scores, factorized **R** using WALS, and measured the resulting dot-products against the Gold Standard dataset, shown in Figures 7, 8 in green. Since WALS produces real-valued predictions, we chose the 0.8 prec. threshold, the comparable precision of the CS and UGC methods, and measured the recall at that threshold with increasing UGC over time.

Note that some of the 〈*p, o*〉 pairs in the Gold set were in the training set, however the *labels* used in the training matrix may be different than Gold, making it a fair comparison. As in the previous experiments we broke the dataset into sets representing the first *n* ∈ [1, 24] months of collected user responses. WALS clearly improves over UGC.

#### 6.3.5. CS+UGC

While 11 months is the intersection point of the metric values for CS and UGC independently, the CS data is supposed to complement as well as jump-start the knowledge acquisition. We tested the role of CS over time using a simple “CS as default” combination, shown in Figures 7, 8 as CS+UGC, in which the UGC label is used if present, and the CS label is used if not. This line tracks the improvement in recall over time from UGC collection, while jump starting at the recall of CS. This is a clear demonstration of our core research hypothesis.

Of particular interest is the comparison of WALS(UGC) with CS+UGC. The former does eventually surpass the latter for shopping after roughly 18 m (Figure 7), but the CS+UGC combination is a strong contender from an extremely simple method. This is again clear evidence of our core hypothesis. However, for dining the story is not so clear, as the WALS(UGC) very quickly reaches near-parity with CS+UGCafter only 5 months, and starts to improve over it in the 11th month of UGCcollection (Figure 8). The reason for this is not entirely clear, the dining matrix is smaller than shopping—the number of restaurants and the number of dishes are both smaller—meaning the same amount of data collection is a higher part of the total matrix. There may be something slightly easier about the restaurant problem as well—restaurant menus tend to be much smaller than the number of products sold in most stores. Perhaps most importantly, for the early part of gathering shopping UGC, we did not have the crowd sense data to guide the collecting, that was available after 6 months, whereas for dining we collected the crowd sense data first and it guided the collection from the start.

Other ways of filling the initial training matrix **R** by combining CS, UGC, and WebIE signals in various ways were tried but not included as they do not outperform WALS(UGC). Of note is that the CS signal does not work well with WALS, since it effectively does what WALS itself should do with enough data - filling in giant portions of the matrix with default values. Other machine learning approaches are certainly possible, indeed the launched *local search* system uses a deep neural network with many more features that are beyond the scope of this paper, and measured at the scale of the web. The three signals reported here are very signifant features of that system, and the full system improves significantly over search alone.

## 7. Related Work

The core of this work is overcoming a *knowledge acquisition* bottleneck in acquiring data reflecting the availability of products at millions of brick and mortar stores worldwide. The approach of harnessing class-level knowledge to infer instance-level knowledge is based on a long standing idea in knowledge engineering, dating back at least as far as Minsky (1974). Other methods in the formal *knowledge representation* (KR) field have never scaled to the level necessary for our problem, nor have they considered the problem of how to acquire distributions instead of discrete facts.

*Information Extraction* (IE) methods perform knowledge acquisition of real-world entities from web text, and are discussed in Zang et al. (2013). Martínez-Rodríguez et al. (2020) present a survey of IE techniques for populating semantic structures, e.g., entity extraction and linking. In the context of shopping, research has mainly focused on product information extraction, e.g., crawling the Web for offers to maintain product catalogs as in Nguyen et al. (2011) and Qiu et al. (2015a), extracting product specifications and attributes as with Kannan et al. (2011), Qiu et al. (2015b), Zheng et al. (2018), and Wang et al. (2020), and IE methods for building product knowledge graphs such as Dong (2020) and Xu et al. (2020). Our paper defines a method for linking these already defined entities similar to Dong (2020), incorporating product and store taxonomy knowledge.

*Knowledge Base Completion* (KBC) is the problem of inferring missing entities and/or relations in an existing knowledge graph based on existing ones, such as via link prediction as in Bordes et al. (2013) or from a combination of sources such as Riedel et al. (2013). Our product × store category matrix (Figure 4) is inspired by the item-based collaborative filtering matrix introduced in recommender systems found in Sarwar et al. (2001) and Ekstrand et al. (2011), and we leverage a well-known collaborative filtering approach introduced in Koren et al. (2009) for KBC to demonstrate the additional power of inference on our knowledge graph.

We use a knowledge graph as the basic representation and, like most well known KGs, employ no general-purpose reasoning; hence, any inference we do must be defeasible. The most relevant KR area would be reasoning with defaults (e.g., Reiter, 1978; Lang, 2000), as our CS+UGC baseline mechanism for combining $\langle c_{p},c_{o}\rangle$ with $\langle i_{p},c_{o}\rangle$ pairs treats the first as a default and the second as an override. Beyond this simple combination strategy, which was first proposed in Quillian (1967), more sophisticated combinations of CS+UGC with other forms of evidence are done using optimizations from machine learning. The full *local shopping system* uses many signals, of which we've described only three, that are combined using a deep neural network that optimizes the prediction of observed labels for many billions of $\langle i_{p},c_{o}\rangle$ pairs. While we exploit the taxonomies in $C_{P}$ and especially $C_{O}$ to optimize the selection of class-level pairs to acquire from workers as discussed in Lees et al. (2020), taxonomy-based reasoning was only used for negative associations. This negative inheritance was first observed by Deng et al. (2014).

IE and KBC techniques have advanced the state-of-the-art in capturing human knowledge in machine-readable form, but there is still the need for human curation and *crowdsourcing*. Important milestones for crowdsourcing knowledge acquisition at scale are Wikidata (Bollacker et al., 2008) and Freebase (Vrandečić and Krötzsch, 2014), where the crowd defines or curates real world entities and some relationships between them, typically driven by Wikipedia. With respect to KBC, Revenko et al. (2018) propose a method for crowdsourcing categorical common sense knowlegde from nonexperts for adding new relationships between nodes in the graph and ensuring consistencey with existing relations. However in all these sources, Taylor (2017) has pointed to the sparsity of graph edges expressing relations between the class-level nodes. Our work focuses directly on that problem by acquiring both class-level and instance level graph edges, and scaling the latter from the former.

The crowdsourcing approach we propose in this paper is grounded in the theoretical framework of Aroyo and Welty (2013) and Aroyo and Welty (2014), which breaks the constraints of typical methodologies for collecting ground truth, showing disagreement is a necessary characteristic of annotated data; when interpreted correctly, Dumitrache (2019) showed it can make evaluation of machine learning models more attuned to real-world data.

The immense body of research on common sense and crowdsourcing has directly influenced our work. The UGC and Crowd Sense tasks drew on our knowledge of Games-with-a-purpose such as Verbosity for collecting common sense facts (von Ahn et al., 2006), Common Consensus for gathering common sense goals (Lieberman et al., 2007), GECKA for common sense knowledge acquisition (Cambria et al., 2016), Concept Game for verifying common sense knowledge assertions (Herdagdelen and Baroni, 2010), the FACTory Game for facts verification (Lenat and Guha, 1989) and many others. Rodosthenous and Michael (2019) refer to common sense as “knowledge about the world" and propose a hybrid (machine and human tasks) workflow to gather general common sense knowledge rules.

*Active learning* investigates efficiency for acquisition and learning when acquiring training data for ML models. In essence, the early stages of KG acquisition strongly represent the exploration side of the *exploration vs. exploitation* tradeoff introduced by Bondu et al. (2010). ML models during exploration do not have enough knowledge of the space to be able to offer reliable judgements as to which items (in this case, $\langle i_{p},c_{o}\rangle$ pairs) to acquire labels for. As noted in the Section 6.1, class-level pairs can serve as a guide for recognizing obvious $\langle i_{p},c_{o}\rangle$ pairs that likely do not need labels, and conversely, high-disagreement pairs are very likely to have instances that do. Thus the $\langle c_{p},c_{o}\rangle$ pairs can serve to stratify the $\langle i_{p},c_{o}\rangle$ space, and make the job of active learning easier by narrowing down their targets. In Section 4 we discussed using these *possible* class-level pairs to guide sampling for UGC.

The problem of mining “interesting” negative statements from Wikidata was investigated in Karagiannis et al. (2019), Arnaout et al. (2020), and Arnaout et al. (2021), which in principle could be used to supplement our active learning strategies for selecting difficult training examples to improve the model. Specifically, these could be combined with the obvious (positive and negative) class-level pairs to find exceptions at individual stores, e.g., a grocery store that does not sell milk or that sells certain tools. Our approach would be slow to find such exceptions, since we don't ask users and would need other sources of evidence used by the larger production syste (e.g., a web page, a user review, etc.). *Peer-based detection*, which compares triples with other triples that share entities in the same category, is similar in spirit to collaborative filtering (CF) though they did not compare experimentally against a CF method such as WALS. *Pattern-based detection*, presented in Karagiannis et al. (2019) and Arnaout et al. (2020) seems better suited for mining (negative) trivia than for product availability, since it is unlikely many online users write about e.g., why supermarkets don't sell asphalt.

Perhaps the most similar crowdsourcing work to ours studies the problem of approximating aggregation queries presented in Trushkowsky et al. (2013), such as “How many restaurants in San Francisco serve scallops?” While this approach works well for estimating counts, clearly it does not scale for KBC.

## 8. Conclusions

The CrowdSense approach was an integral part of a successful worldwide launch of local search results to queries for products or dishes, overlaid on Google Maps, as shown in Figure 1. Due to the complexity and scope of the deployed project, we focused on the real-world knowledge acquisition aspect of the work, and presented a few simplified experiments that demonstrate how the acquired class-level knowledge can be used for KBC at the instance level. These experiments may seem over-simplified, but they accurately capture the impact of the three-tiered crowdsourcing approach on the deployed product, in particular the rapid jump-start of the place-offering edges in the knowledge graph.

To achieve these results, we augmented an existing knowledge graph of most stores and restaurants on earth, their categories, dishes and a product taxonomy, by adding place to product and place to dish edges. We combined web-based information extraction (WebIE) and direct user observations collected over 2 years (UGC) with a novel collection of class-level 〈*store, offering*〉 pairs from the crowd (CS), which were inferred to the instance-level based on class membership. In 2 weeks of data collection we achieved a recall of 0.24 at 0.80 precision against gold standard instance-level labels for shopping, and 0.21 for dining. The class-level data for shopping combined with WebIE to achieve 0.35 recall, which was the recall of a WALS model with 18 months of UGC input. For dining the same combination also produced 0.34 recall, which was the WALs recall for 11 months of UGC. We conclude that the Crowd Sense approach uses human common sense knowledge to *rapidly jump start* the kind of generalization that ML systems are good at with a lot of data. This has implications for practical ML and Human Computation.

Our class-level crowdsourcing results show that the disagreement in categorical knowledge collected from the crowd can indicate the distribution of that knowledge at the instance level, rather than assuming the class-level associations are universally true: in other words, if 80% of raters say “Grocery stores sell oat milk,” then ~80% of grocery stores sell oat milk. These results held also for dishes at restaurants.

The taxonomy of products was used to guide the sampling of class-level pairs in a way that helped us address the sparsity of the $C_{P}\times C_{O}$ space, and only the *negative* class-level attributes were accurate when inferred to more specific categories, as in Deng et al. (2014), as opposed to the more traditional view that positive attributes are “inherited.”

We found the categorical pairs which were rapidly acquired were extremely useful in guiding the collection of instance-level labels, since we did not have to ask users about obviously available or unavailable products—this has implications for active learning, and held also for dining.

We expected the class-level ratings we acquired from a small, international, pool of paid raters, to show bias toward ratings coming from the same country of a restaurant. In other words, we expected class-level ratings from Indian raters to have lower error for restaurants in India than class-level ratings from raters in other countries. This turned out to only be true for Japan, and for all other countries it was the opposite. This may tell us something about the way the place categories model the real world, more investigation is required.

We believe Crowd Sense is a general technique for knowledge acquisition that can provide a rapid jump-start to the process by acquiring more general, common-sense defaults as a first step, while more precise but time-consuming acquisition (i.e., at the instance level) proceeds over time. We have shown that the original local shopping idea, first presented in Welty et al. (2021), can generalize to other establishment domains with similar gains, in this case dining, and we have considered many other bipartite problems that meet the basic requirement that there is a strong, common-sense understanding of the relation at the categorical level, for example:

- *Dish contains ingredient*. Dishes have associated recipes and a strong notion of taxonomy^8^, and many ingredient associations are ridiculous at a class level, such as Apple Pie and Curry.
- *Cuisine includes dish*. Dishes are also associated with cuisines, a pairing that could be useful for recipe datasets, and understanding menus. Many cuisines are regional, introducing a different kind of partial order (containment rather than generalization, see Guarino and Welty, 2009) on one side of the bipartite relation.
- *Wildlife inhabiting a region*. Several NGOs track wildlife populations through remote cameras and citizen science collection of photos, and identify animals using automatic methods.^9^ Such methods would benefit from large scale understanding of obvious negatives (tigers are not found in Africa). Like cuisines, this involves treating locations as a partial order based on containment, and the Linnaean taxonomy for animals is well established.
- *Animal has body part*. In the early days of AI, much ink was spilled on modeling defaults and exceptions such as “Elephants have trunks” and “Humans have two legs.” This work was summarized nicely in Brachman (1985). Modern AI systems do not use this information and rely on the formation of embeddings that bely human understanding, but such systems have been shown in Aroyo and Paritosh (2021) to make “silly” categorical mistakes. An approach that forces large models to form meaningful intermediate representations such as parts of the body, as described by Hinton (2021), could avoid silly mistakes with this form of common sense curation.
- *Company owns patent*. Finding patents is a difficult search task that continues to be a focus of AI systems. While these systems do not generally lack data, they do often suffer from silly mistakes, as image understanding systems do, which reflect a lack of common sense. Adding categorical associations such as, “Tech companies do not own pharmaceutical patents” would eliminate some of these mistakes.

**Figure 9:**
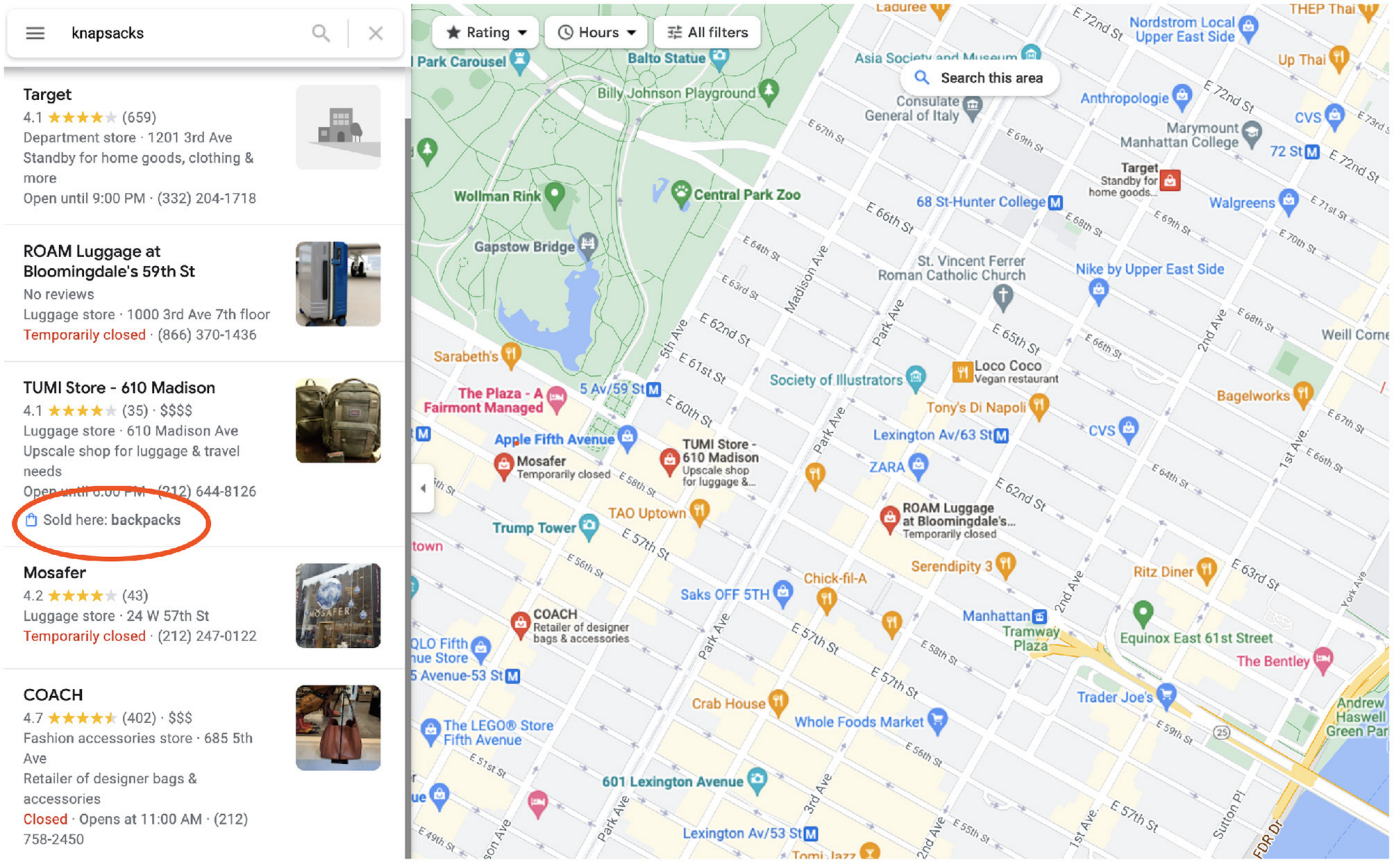
CrowdSense search results in NYC for knapsacks.

To see CrowdSense at work, type the name of a product or dish into Google Maps (or Google Search). Results that say “Sold here: *product*” come from the data we published (see Figure 9, as opposed to “In stock” (merchant feeds) and “Webpage says.” Anyone with a Google account can participate in UGC (user generated content) acquisition. Users with location tracking turned on (so that maps knows what places the user has visited^10^) can navigate to the “contribute” tab that allows them to rate and leave reviews, as well as review facts and answer the yes/no questions regarding locations they have visited.

## Data Availability Statement

The original contributions presented in the study are included in the article/supplementary materials, further inquiries can be directed to the corresponding author/s. The categories of places and products are available and included in the article, and the rest of the data discussed in this paper is not, as it is proprietary data that drives Google Maps.

## Author Contributions

CW led the project. CW and FK wrote most of the article. All authors contributed experimental results and background research.

## Conflict of Interest

All authors were employed by Google Research.

## Publisher's Note

All claims expressed in this article are solely those of the authors and do not necessarily represent those of their affiliated organizations, or those of the publisher, the editors and the reviewers. Any product that may be evaluated in this article, or claim that may be made by its manufacturer, is not guaranteed or endorsed by the publisher.

## Footnotes

^1^ https://support.google.com/merchants/answer/9825611?hl=en

^2^ https://www.forbes.com/sites/jiawertz/2018/05/17/how-brick-and-mortar-stores-can-compete-with-e-commerce-giants/#2019f5a23cc0

^3^ https://support.google.com/business/answer/3038177/#categories

^4^ https://bayareawebsitedesigner.com/gmb-categories/

^5^ https://www.google.com/basepages/producttype/taxonomy.en-US.txt

^6^ https://feedonomics.com/google_shopping_categories.html

^7^Collection continues, these windows were used for this paper.

^8^e.g., https://www.wikidata.org/wiki/Wikidata:WikiProject_Food/Taxonomy.

^9^Examples include wildlifeinsights.org and inaturalist.org.

^10^See https://support.google.com/local-guides/answer/6225846.
